# Transcriptome and Metabolome Analyses of *Thitarodes xiaojinensis* in Response to *Ophiocordyceps sinensis* Infection

**DOI:** 10.3390/microorganisms11092361

**Published:** 2023-09-21

**Authors:** Miaomiao Li, Jihong Zhang, Qilian Qin, Huan Zhang, Xuan Li, Hongtuo Wang, Qian Meng

**Affiliations:** 1 College of Basic Medicine, Shaanxi University of Chinese Medicine, Xianyang 712046, China; 2 State Key Laboratory of Integrated Management of Pest Insects and Rodents, Institute of Zoology, Chinese Academy of Sciences, Beijing 100101, Chinazhanghuan@ioz.ac.cn (H.Z.);

**Keywords:** integrated omics analysis, fungus–host interaction, immune defense, lipid metabolism, amino acid

## Abstract

*Ophiocordyceps sinensis* exhibits more than 5 months of vegetative growth in *Thitarodes xiaojinensis* hemocoel. The peculiar development process of *O. sinensis* has been elucidated through morphological observation and omics technology; however, little information has been reported regarding the changes that occur in the host *T. xiaojinensis*. The RNA sequencing data showed that when *O. sinensis* blastospores were in the proliferative stage, the greatest change in the infected larval fat body was the selectively upregulated immune recognition and antimicrobial peptide genes. When *O. sinensis* blastospores were in the stationary stage, the immune pathways of *T. xiaojinensis* reverted to normal levels, which coincides with the successful settlement of *O. sinensis*. Pathway enrichment analysis showed a higher expression of genes involved in energy metabolism pathway in this stage. Metabolomic analyses revealed a reduction of amino acids and lipids in hemolymph, but an upregulation of lipids in the fat body of the host larvae after *O. sinensis* infection. We present the first transcriptome integrated with the metabolome study of *T. xiaojinensis* infected by *O. sinensis*. It will improve our understanding of the interaction mechanisms between the host and entomopathogenic fungi, and facilitate future functional studies of genes and pathways involved in these interactions.

## 1. Introduction

The larvae of the ghost moth *Thitarodes xiaojinensis* (Tu.) [1,2] (Lepidoptera: Hepialidae) are hosts of *Ophiocordyceps sinensis* (Berk.) (Hypocreales: Ophiocordycipitaceae) [3,4]. The fungus–caterpillar complex, called Chinese cordyceps, has a long history of use as a traditional Chinese medicine for the treatment of many diseases [5,6]. Fungal conidia can adhere to the host cuticle, germinate penetration pegs and proliferate in the form of blastospores in the host hemocoel [7]. After successful invasion into the host hemocoel, *O. sinensis* blastospores were divided into proliferative (BP) and stationary (BS) stages according to whether they were actively proliferating. The BS could accumulate up to ~6 × 10^8^ blastospores/mL and then turn into prehyphae, followed by germination of hyphae in the host’s last instar larvae. The infection lasts at least 5 months until the host larvae are killed [8]. The unique development process of *O. sinensis* in ghost moth larvae and insect immunological responses during the early phase of infection (within several days) has been well described [7,8,9]. However, studies on the immune and metabolic changes of *T. xiaojinensis* during the long-term interaction between the host and *O. sinensis* are lacking.

Insects rely on the innate immunity made up of cellular and humoral immunity to defend against microbial infection. Cellular immune responses include phagocytosis, encapsulation and nodulation. They are elicited through the aggregation of hemocytes, and produce immune factors to eliminate pathogens via lysis and/or melanization. Humoral immune responses mainly consist of the production of antimicrobial peptides (AMPs), reactive oxygen and nitrogen species and activation of the prophenoloxidase system [10,11,12]. Innate immunity constitutes a complex and interconnected network. It can protect insects from invasion by microbes, but it is not omnipotent [9,13,14]. Entomopathogenic fungi, such as *Beauveria bassiana* (Hypocreales: Cordycipitaceae) and *Metarhizium anisopliae* (Hypocreales: Clavicipitaceae), can integrate the strategies of nutrient deprivation, toxin production and inhibition of host immune responses to kill insects in as quickly as two weeks [15,16,17]. However, *Ophiocordyceps sinensis* can peacefully coexist with ghost moths for approximately half a year or more, and the feeding behavior and movement of larvae are not affected [8]. Previous studies have demonstrated that the immunity-related genes of *T. xiaojinenesis* were capable of a rapid response (less than 72 h) to an *O. sinensis* challenge. *T. xiaojinenesis* larvae developed tolerance to the fungus after prolonged infection (1 year) [18]. It was unknown when this change occurred. It is not known what happens to the host immune response during the intermediate stages of infection, especially during the long period of the BP and BS stages. These two stages account for most of the survival period of the infected host, and yet this has not been investigated.

Fungi can modify insect host lipid metabolism [15,17,19,20]. Lipids are a class of organic compounds that include fatty acids and their derivatives. Triglycerides (TAGs) stored in fat body cells are the main energy resource of insects [21]. Phospholipids serve as one of the main components of a cell membrane. Some lipids may play roles in signal transduction. The levels of various lipids in hosts are increased in the case of fungal infected insects, which may relate to the elevated biomolecular demands for energy production and storage, membrane repair and signaling pathway intermediates [22,23].

Transcriptomics play a role in analyzing the gene expression pattern and function [24], while metabolomics assist in identifying metabolites changes in biological events [25]. Metabolites are the signature for the downstream of gene and protein expression linking the genotypes and phenotypes in the organism [26]. Combined metabolome and transcriptome analyses can detect genes, pathways and metabolites related to a physiological response with high sensitivity and validity. Therefore, we applied these two methods to reveal the molecular changes in *Thitarodes xiaojinensis* infected by *Ophiocordyceps sinensis*.

Insect hemolymph is a depository of nutrients, energy and metabolic intermediates, not only for insects, but also for the entomopathogenic fungi [27,28]. Insect fat body plays important roles in immunity, metabolism and nutrient storage [29]. Here, integrated transcriptome and metabolome analyses of the *Thitarodes xiaojinensis* hemolymph and fat body were carried out to elucidate the mechanism of *T. xiaojinensis* in response to *Ophiocordyceps sinensis* infection during the blastospore stage. This study will improve our understanding of the interaction mechanisms between the host and entomopathogenic fungi, and reveal the mechanism of the long-term coexistence of host and fungus.

## 2. Materials and Methods

### 2.1. Experimental Fungi

Information on *Ophiocordyceps sinensis* was provided beforehand [8]. Briefly, *O. sinensis* was isolated from fresh Chinese cordyceps in Xiaojin Country, Sichuan province, China, and was cultured and maintained on potato dextrose agar at 18 °C.

### 2.2. Experimental Insects

Information on *Thitarodes xiaojinensis* was provided beforehand [8]. *T. xiaojinensis* were collected from Xiaojin county, Sichuan province, China, and subsequently reared in the laboratory for several generations. The fifth instar ghost month larvae were inoculated by injection with a glass capillary loaded with 5 μL diluted suspensions containing 3 × 10^6^ blastospores/μL. Control larvae were similarly treated with Ringer’s buffer [8.05 g NaCl (Solarbio Science & Technology, Beijing, China), 0.42 g KCl (Solarbio) and 0.18 g CaCl_2_ (Solarbio) per liter].

### 2.3. Collection of Larvae Samples

The larvae samples (including hemolymph and fat body) were collected as Tx-BP when the fungus entered the BP stage (sampled at one month after *Ophiocordyceps sinensis* infection) [30]. MOCK1, set as the control for Tx-BP, were the samples collected from the Ringer’s-injected larvae, developing into the same instar as the larvae infected with BP. The larvae samples were collected as Tx-BS when the fungus entered BS stage (sampled at four months after *O. sinensis* infection) [30]. MOCK2, set as the control for Tx-BS, were the samples collected from the Ringer’s-injected larvae, developing into the same instar as the larvae infected with BS.

### 2.4. Quantitative Analysis of TAGs

A TAG content determination kit (Solarbio) was utilized to assay the total TAG content of the samples according to the manufacturer’s protocol [31]. Briefly, the fat body samples were collected and reacted with the reagents provided. The optical density was measured spectrophotometrically at a wavelength of 420 nm. Every group contained three or four individual larvae and were technically measured in triplicate.

### 2.5. RNA Extraction, Library Preparation and RNA-Sequencing

Each group, namely, Tx-BP, MOCK1, Tx-BS and MOCK2, contained three biological replicates. Each biological replicate was collected from three individual larvae. For RNA-sequencing, we collected samples from diverse *Thitarodes xiaojinensis* fat bodies (Tx-BP, MOCK1, Tx-BS and MOCK2) as previously described [30]. The complementary DNA (cDNA) libraries were constructed and sequenced by Shanghai Majorbio Biopharm Technology Co., Ltd. (Shanghai, China). After quantification by TBS380 (Invitrogen, Carlsbad, CA, USA), the library was sequenced by the Illumina HiSeq xten/NovaSeq 6000 sequencer (Illumina, San Diego, CA, USA) using the paired-end method, 2 × 150 base pair (bp) read length.

### 2.6. Gene Expression Profiling and Data Analysis

After trimming, quality control of the raw paired-end reads was performed by SeqPrep (https://github.com/jstjohn/SeqPrep, accessed on 30 July 2022) and Sickle (https://github.com/najoshi/sickle, accessed on 30 July 2022) with default parameters. Gene abundances were quantified by RSEM (v.1.3.1, http://deweylab.biostat.wisc.edu/rsem/, accessed on 30 July 2022) [32] and present as the transcripts per million reads (TPM). Differentially expressed genes (DEGs) identified between two different samples were calculated using DESeq2 (v.1.24.0) in R [33]. *p* values in the analysis of the false discovery rate (FDR) were adjusted according the Benjamini and Hochberg method [34]. Genes with a|Log2 (FoldChange)| ≥ 1 and a FDR ≤ 0.05 in a comparison were identified as DEGs. Gene Ontology (GO) functional enrichment and Kyoto Encyclopedia of Genes and Genomes (KEGG) pathway analysis were carried out by Goatools (https://github.com/tanghaibao/Goatools, accessed on 30 July 2022) and KOBAS (http://kobas.cbi.pku.edu.cn/home.do, accessed on 30 July 2022), respectively [35]. 

### 2.7. Metabolite Detection

For the metabolomic analysis, six biological replicates were detected for each group. Samples for each replicate were collected from three individual larvae. Liquid chromatography–tandem mass spectrometry (LC-MS/MS) analyses of hemolymph samples were performed as previously described [30]. LC-MS/MS analyses of fat body samples were as follows. Chromatographic metabolites separation was carried out on a Thermo UHPLC system equipped with an ACQUITY UPLC HSS T3 (Waters Corporation, Milford, MA, USA) column (100 mm × 2.1 mm, 1.7 µm). The mobile phases consisted of solvent A (0.1% formic acid in water: acetonitrile = 0.95:0.05, *v*/*v*) and solvent B (0.1% formic acid in acetonitrile: isopropanol: water = 0.475:0.475:0.05, *v*/*v*/*v*). To equilibrate the systems, the solvent gradient changed according to the following conditions: from 0 to 2 min, 100% (A): 0% (B) to 75% (A): 25% (B); from 2 to 9 min, 75% (A): 25% (B) to 0% (A): 100% (B); from 9 to 13 min, 0% (A): 100% (B) to 0% (A): 100% (B); from 13 to 13.1 min, 0% (A): 100% (B) to 100% (A): 0% (B); and from 13.1 to 16 min, 100% (A): 0% (B) to 100% (A): 0% (B). During the analysis, all samples were stored at 4 °C, while the column was maintained at 40 °C. The sample was injected in the volume of 2 μL with the flow rate of 0.4 mL/min. A thermo UHPLC-Q Exactive Mass Spectrometer equipped with an electrospray ionization (ESI) source operating in either positive or negative ion mode was used to collect the mass spectrometric data. After UPLC-TOF/MS analyses, peak detection and alignment of the raw data were performed via Progenesis QI 2.3 (Nonlinear Dynamics, Waters Corporation, Milford, MA, USA). Mass spectra features of the metabolites were detected by using the accurate mass, MS/MS fragment spectra and isotope ratio difference through searching the Human Metabolome Database (HMDB) (http://www.hmdb.ca/, accessed on 30 July 2022) and the METLIN database (https://metlin.scripps.edu/, accessed on 30 July 2022).

### 2.8. Differential Metabolite Analysis

Principal component analysis (PCA) using an unsupervised method was applied to visualize general clustering, trends or outliers of each group. To evaluate the overfitting of the model, 200 permutation tests were executed in the partial least squares discriminate analysis (PLS-DA) model. Metabolites differences were identified on the following criterion: variable importance in the projection (VIP) > 1, *p* value < 0.05 and fold change (FC) ≥ 2 or ≤0.5. Each stage of the sample was mixed with an equal amount, serving as the quality control (QC)sample.

### 2.9. Quantitative Real-Time Polymerase Chain Reaction (qRT-PCR)

RNA isolation and qRT-PCR were performed as previously reported [18]. All of the primers used in this study are shown in Supplementary File S1: Table S5. Relative gene expression levels were determined using the 2^−ΔΔCT^ method [36]. The Mock group was used for normalization and the ribosomal protein S3 (rpS3) was set as the internal reference gene. All of the data were obtained from three biological replicates, with three technical replicates each, and statistically analyzed in GraphPad Prism 7 (GraphPad Software, Boston, MA, USA, www.graphpad.com, accessed on 30 July 2022) or R (A Language and Environment for Statistical Computing, Vienna, Austria, www.r-project.org, accessed on 30 July 2022) using one-way analysis of variance (ANOVA), followed by Tukey’s test (*p* < 0.05).

## 3. Results

### 3.1. The Host Body Weight and TAG Concentration Increased in the Fat Body after Ophiocordyceps sinensis Infection

**Figure 1:**
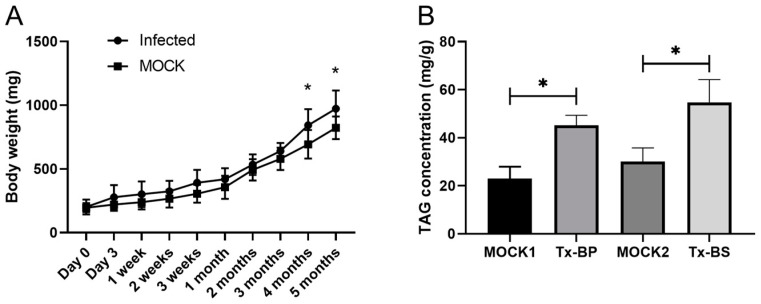
Increase in *Thitarodes xiaojinensis* whole body weight and TAG concentration after *Ophiocordyceps sinensis* infection. (**A**) Whole body weight growth curves of *T. xiaojinensis* fifth larvae after infection with *O. sinensis*. (**B**) TAGs concentrations in infected and control larvae. The bars represent the mean ± SEM of values from three technical repeats, and the asterisks above the bars indicate significant differences in the *t*-test (* *p* < 0.05). Tx-BP, *T. xiaojinensis* samples collected when *O. sinensis* entered BP stage; MOCK1, the control for Tx-BP; Tx-BS, *T. xiaojinensis* samples collected when *O. sinensis* entered BS stage; MOCK2, the control for Tx-BS.

To determine the effect of *Ophiocordyceps sinensis* infection on host growth, we recorded the whole body weight of each infected and mock larvae from the first day of injection. Body weight was continuously higher in the *O. sinensis* group than in the mock group, and was significantly higher after the 4-month period (Figure 1A). The infected host showed significantly higher concentrations of TAGs in the fat body than the mock larvae at both the BP and BS stages (Figure 1B).

### 3.2. Transcriptome Profiling

To describe the dynamic gene expression patterns in the fat body between infected and uninfected *Thitarodes xiaojinensis*, 12 cDNA libraries were constructed using samples from four groups, namely, Tx-BP (*T. xiaojinensis* samples collected when *Ophiocordyceps sinensis* was in the BP stage), MOCK1 (the control for Tx-BP), Tx-BS (*T. xiaojinensis* samples collected when *O. sinensis* was in the BS stage), and MOCK2 (the control group for Tx-BS), with three biological replicates for each group. A total of 68,777 unigenes were obtained from 12 cDNA libraries. Their sequencing quality is summarized in Supplementary file S1: Table S1. The mean length and N50 of the unigenes were 1025 bp and 1832 bp, respectively. In total, 20,045, 15,257, 27,726, 27,409, 18,836 and 22,115 unigenes were annotated in the GO, KEGG, Clusters of Orthologous Groups of proteins (COG), NCBI nonredundant protein sequences (NR), a manually annotated and reviewed protein sequence database (SwissProt) and the Protein family (Pfam) databases, respectively (Supplementary file S1: Table S2). Principal component analysis (PCA) assigned samples into four groups, referred to as MOCK1, MOCK2, Tx-BP and Tx-BS (Supplementary file S2: Figure S1). Tx-BP and MOCK1 were closely related, suggesting that the two groups had more similar expression patterns.

To gain insights into global transcriptional changes in *Thitarodes xiaojinensis* larvae infected by *Ophiocordyceps sinensis*, pairwise comparisons were performed between control and infected groups to identify DEGs. A summary of the DEGs between the two groups is provided in Supplementary file S1: Table S3. A total of 3446 DEGs were identified in the Tx-BS vs. MOCK2 group. It included 2001 upregulated and 1445 downregulated genes. Fewer DEGs were detected in the Tx-BP vs. MOCK1 group, including 472 upregulated and 387 downregulated genes (Supplementary file S2: Figure S2).

### 3.3. Gene Responses to Ophiocordyceps sinensis Infection

**Figure 2:**
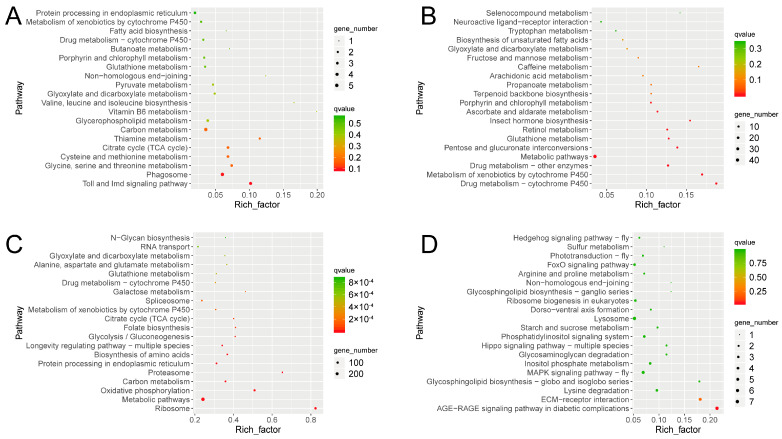
KEGG significant enrichment analysis for differentially expressed genes (DEGs). (**A**) KEGG significant enrichment analysis for the upregulated genes in Tx-BP compared with MOCK1. (**B**) KEGG significant enrichment analysis for the downregulated genes in Tx-BP compared with MOCK1. (**C**) KEGG significant enrichment analysis for the upregulated genes in Tx-BS compared with MOCK2. (**D**) KEGG significant enrichment analysis for the downregulated genes in Tx-BS compared with MOCK2. Tx-BP, *T. xiaojinensis* samples collected when *O. sinensis* entered BP stage; MOCK1, the control for Tx-BP; Tx-BS, *T. xiaojinensis* samples collected when *O. sinensis* entered BS stage; MOCK2, the control for Tx-BS.

To analyze the potential functions of all identified DEGs and the corresponding pathways, GO and KEGG enrichment analyses were performed [37,38]. For the Tx-BP vs. MOCK1 group, KEGG enrichment analysis showed that the Toll and immune deficiency (IMD) signaling pathway and phagosome pathway were significantly enriched for the upregulated genes (Tx-BP vs. MOCK1). The drug metabolism cytochrome P450 and metabolism of xenobiotics by cytochrome P450 were significantly enriched for the downregulated genes (Figure 2A,B). For the Tx-BS vs. MOCK2 group, upregulated genes in the Tx-BS were mainly associated with ribosomes, metabolic pathways and oxidative phosphorylation pathways. The downregulated genes were mainly enriched in the advanced glycation end products (AGEs) that interact with the receptor for the AGEs (AGE–RAGE) signaling pathway in diabetic complications and extracellular matrix (ECM)-receptor interactions (Figure 2C,D). 

We also used the GO classification to analyze putative functions of the *Ophiocordyceps sinensis*-induced DEGs in different stages. GO analysis showed that DEGs were classified into three major functional categories: biological process, cellular component and molecular function based on the criteria of *p* value ≤ 0.05. Figure S3 shows the top 30 GO terms, with the most abundant DEGs in the three categories in two comparisons. For the BP vs. MOCK1 group, peptidoglycan binding, peptidoglycan muralytic activity, humoral immune response, immune system process and defense response to other organisms were observed as the top five most enriched GO terms. This is consistent with the KEGG enrichment results of active immunity in the Tx-BP stage. For the BS vs. MOCK2 group, the top five most enriched GO terms were purine nucleoside triphosphate biosynthetic, purine ribonucleoside triphosphate biosynthetic, purine nucleoside triphosphate metabolic, purine ribonucleoside triphosphate metabolic process and monovalent inorganic cation transport, which is consistent with the KEGG results of active energy metabolism in the Tx-BS stage. Generally speaking, the GO and KEGG analyses provided an overview of the functions of the DEGs, and the pathways described above might play an important role in the response of *Thitarodes xiaojinensis* to *O. sinensis* infection.

### 3.4. Metabolome Profiling

A nontargeted LC-MS/MS analysis was performed to detect metabolites in the fat body and hemolymph, when *Thitarodes xiaojinensis* was infected with *Ophiocordyceps sinensis* at two time points (the same as the time points at which the samples were collected for the transcriptome analysis). The QC samples were obtained by collecting an equal amount of mixture from the samples at each stage. The tightly clustered QC samples ensured detection stability (Figure S4). All of the QC samples were clustered tightly in each tissue specimen (Figure S4), indicating the good analytical stability and experimental reproducibility of the metabolic profiles. PCA was used to determine the variability in the changes in metabolite contents during the infection process. PCA of all samples in both modes was well intraclustered and interseparated in each tissue specimen (Figure S4), implying the good metabolic repeatability in each group. 

Validation plots showed the stable and credible PLS-DA models. All of the R2Y values were greater than the Q2Y values in the score plots, which also confirmed the stability and credibility of the models (Supplementary file S2: Figure S5). Only the metabolites with a VIP greater than 1.0, a *p* value less than 0.05 and a FC more than 2 or less than 0.5 between the infected and control groups were included in the final data analysis.

### 3.5. Thitarodes Xiaojinensis Metabolite Profiles Were Altered after Ophiocordyceps sinensis Infection 

**Figure 3:**
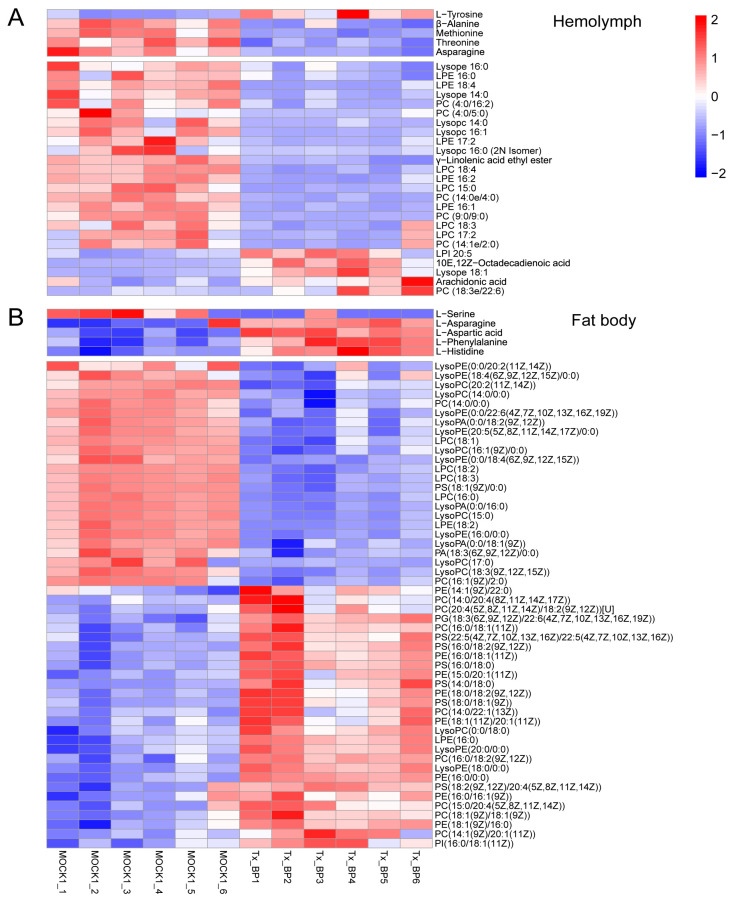
Metabolic profiles across the Tx-BP and MOCK1 groups. The metabolites in the heatmap were identified in the mock and *Ophiocordyceps sinensis*-infected host (Tx-BP) samples using LC-MS/MS analysis. The data are presented as relatively quantitative values of the peak area. Upregulated metabolites are presented in red, whereas downregulated metabolites are shown in blue. (**A**) DEMs detected in the hemolymph samples. (**B**) DEMs detected in the fat body samples. Tx-BP, *Thitarodes xiaojinensis* samples collected when *O. sinensis* entered BP stage; MOCK1, the control for Tx-BP.

We investigated changes in metabolites in the hemolymph and fat body of *Ophiocordyceps sinensis* infected and uninfected larvae. At the Tx-BP stage, the levels of amino acids and lipids decreased in the hemolymph (Figure 3A). Moreover, we found that some differentially expressed metabolites (DEMs) in the fat body were enriched in phospholipid metabolism (Figure 3B). Phospholipase hydrolyses phospholipids to release free fatty acids and lysophospholipids [39]. Lysophospholipids were identified as precursors and metabolites in the de novo biosynthesis of phospholipids [40] and were necessary for the composition and repair of cell membranes. Compared with the control group, the contents of lysophospholipids of lysophosphatidylcholines (LPCs), lysophosphatidic acids (LPAs) and lysophosphatidyl ethanolamines (LPEs) significantly decreased in the infected group. In addition, lysophospholipids of phosphatidylcholines (PCs), phosphatidylethanolamines (PAs) and phosphatidylserines (PSs) accumulated in the infected group (Figure 3B). These results indicate the remodeling of phospholipids and lysophospholipids in the fat body of *Thitarodes xiaojinensis* infected with *O. sinensis* at the BP stage. 

**Figure 4:**
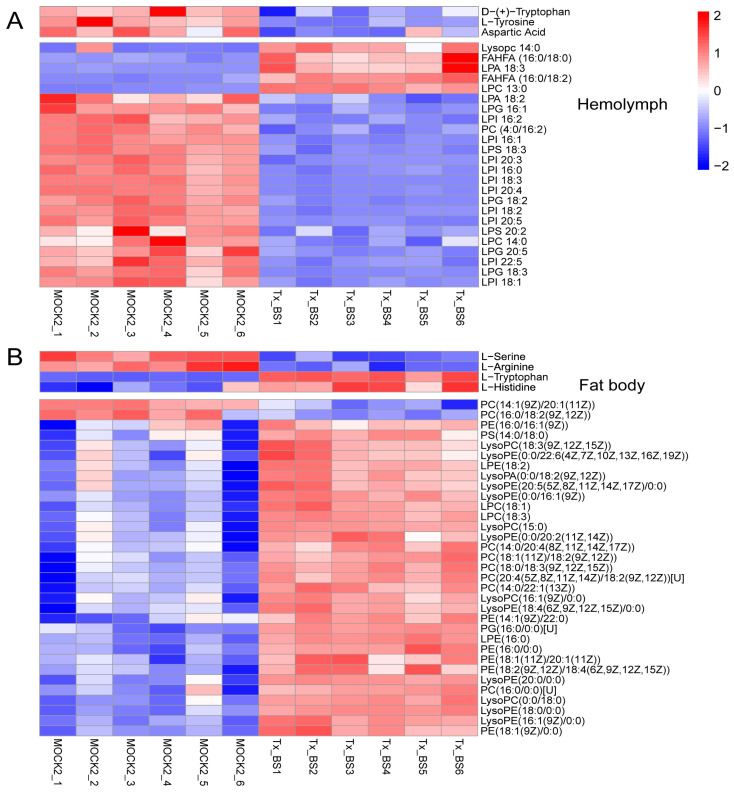
Metabolic profiles across the Tx-BS and MOCK2 groups. The metabolites in the heatmap were identified in the mock and *Ophiocordyceps sinensis*-infected host (Tx-BS) samples using LC-MS/MS analysis. The data are presented as relatively quantitative values of the peak area. Upregulated metabolites are presented in red, whereas downregulated metabolites are shown in blue. (**A**) DEMs detected in the hemolymph samples. (**B**) DEMs detected in the fat body samples. Tx-BS, *Thitarodes xiaojinensis* samples collected when *O. sinensis* entered BS stage; MOCK2, the control for Tx-BS.

After 2–3 months of growth, the blastospore concentration reached a threshold density and developed into BS [8]. The levels of amino acids and lipids decreased in the hemolymph. Compared with MOCK2 larval fat bodies, the fat bodies of infected larvae had significantly higher levels of lipids, including LPA, LPC, LPE, PC, phosphatidyl ethanolamines (PE), PG and PS. However, the levels of amino acids in the fat bodies were not significantly altered in the presence of *Ophiocordyceps sinensis* (Figure 4A,B).

### 3.6. Thitarodes Xiaojinensis Lipid Metabolism Was Affected by Ophiocordyceps sinensis Infection

The lipid metabolism functional group was the vast majority among the differentially accumulated compounds. Glycerolipid is utilized in the metabolism for the generation of lipid energy sources, components of cell membranes and signaling pathway modulators [41]. 

**Figure 5:**
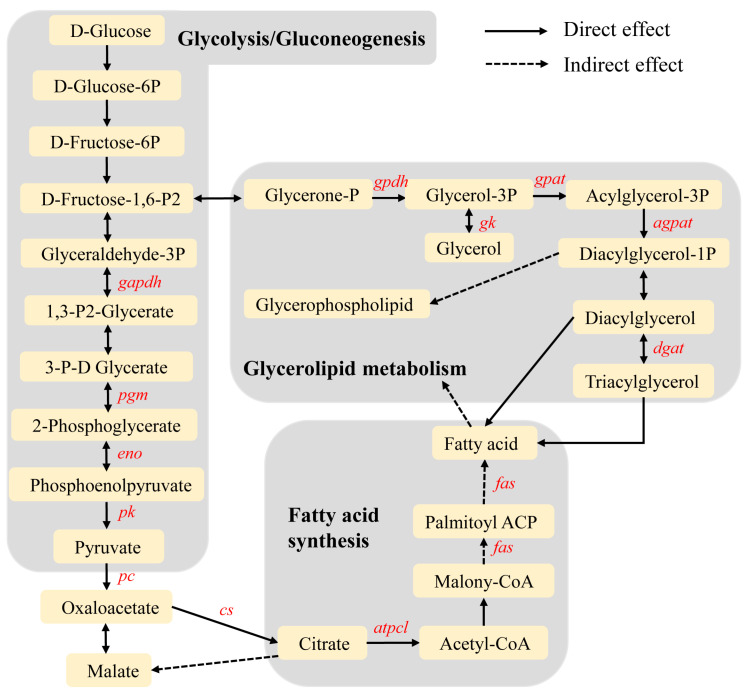
Glycerolipid metabolism and its related pathways. The red italicized text indicates upregulated genes in the fat body in Tx-BS compared with that in MOCK2. Tx-BS, *Thitarodes xiaojinensis* samples collected when *Ophiocordyceps sinensis* entered BS stage; MOCK2, the control for Tx-BS.

Phospholipids form the lipid bilayers of cell membranes due to their amphiphilic structure [42]. Our metabolomic analyses revealed that lipids, especially numerous phospholipids (PCs, PAs, PEs and PSs), increased their concentrations following infection in the *Thitarodes xiaojinensis* fat body. These data support the general view that the amount of stored lipids in infected host fat bodies tends to increase. Based on the transcriptome results, the lipid metabolism pathways associated with the conversion pathway of glucose to glycerolipid were constructed (Figure 5). Glucose first activates the glycolytic pathway that produces pyruvate from glucose. Several genes involved in the pathway, including glyceraldehyde-3-phosphate dehydrogenase (gapdh), phosphoglucose mutase (pgm), enolase (eno) and pyruvate kinase (pk), were upregulated in *T. xiaojinensis* infected at the BS stage. Furthermore, three genes, pyruvate carboxylase (pc), citrate synthase (cs) and ATP citrate lyase citrate (atpcl), which are involved in the conversion of pyruvate to acetyl-CoA, were also upregulated in the infected larvae. In the glycerolipid synthesis pathways, one ortholog of glycerol-3-phosphate dehydrogenase (gpdh), glycerol kinase (gk), glycerol-3-phosphate acyltransferase (gpat), 1-acyl-sn-glycerol-3-phosphate acyltransferase (agpat) and diacylglycerol o-acyltransferase (dgat) was upregulated in the infected larvae (Figure 5). The expression of all the above mentioned genes in the fat body was verified by qRT-PCR (Supplementary file S2: Figure S6). We also detected genes associated with degradation of glycerolipids and fatty acids, such as lipase, glycerol kinase, acyl-CoA synthetase and acyl-CoA dehydrogenase. These genes were upregulated in the fat body of *T. xiaojinensis* infected at the PreHy stage (Supplementary file S2: Figure S7). This suggested an accumulation of the lipids in Tx-BS. It might be used for the later development of both the fungus and the larvae.

### 3.7. Thitarodes Xiaojinensis Oxidative Phosphorylation Pathway Was Affected by Ophiocordyceps sinensis Infection

**Figure 6:**
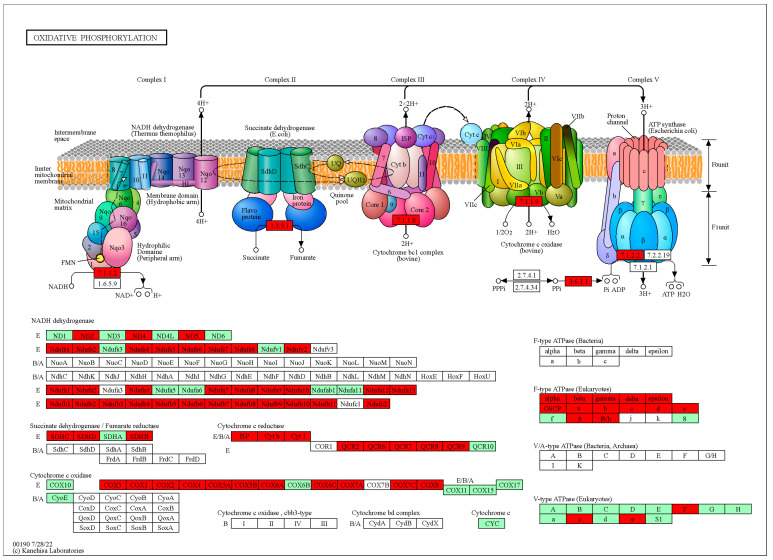
The oxidative phosphorylation pathway. Genes highlighted in red are enriched and upregulated in Tx-BS compared with MOCK2, based on RNA-sequencing data. Tx-BS, *Thitarodes xiaojinensis* samples collected when *Ophiocordyceps sinensis* entered BS stage; MOCK2, the control for Tx-BS.

The oxidative phosphorylation pathway, an energy metabolism pathway, mainly provides ATP as energy [43]. The genes in the oxidative phosphorylation pathway were significantly upregulated in Tx-BS, especially the genes encoding NADH dehydrogenase, succinate dehydrogenase, cytochrome C reductase, cytochrome C oxidase and ATPase (Figure 6).

### 3.8. The Immune Response Effectors Were Upregulated in Tx-BP but Barely Detected in Tx-BS

Immune signal modulation and transduction pathways include Toll, IMD, c-Jun N-terminal protein kinases (JNK), and (Janus kinase/signal transducer and activator of transcription) JAK/STAT, which are triggered by recognition of pathogen-associated molecular patterns (PAMPs) on the microbial surface. Pattern recognition receptors (PPRs) in insects bind to conserved determinants on the surface of microbes and initiate phagocytosis as well as activate proteinase signaling cascades. In our transcriptomic data, two peptidoglycan recognition proteins (PGRP), PGRP-S2 and PGRP-S5, were upregulated in Tx-BP and had no significant change in Tx-BS. Signal modulation molecules and intracellular signal transduction molecules in Toll, IMD, JNK and JAK/STAT pathways showed no significant change (Supplementary file S1: Table S4). Immune response effectors were mainly composed of AMPs, which were primarily produced by the fat body after microbial infection. RNA-seq-based analysis showed a dramatic induction of AMP genes, such as defensin, attacin 4, 5, cecropin 2, 3, 5 and gloverin in Tx-BP. However, the mRNA of the AMPs was barely detected in Tx-BS (Table 1). Overall, the immune system of *Thitarodes xiaojinensis* responded to the infection of *Ophiocordyceps sinensis* infection in the manner of partial activation in Tx-BP and tolerance in Tx-BS.

## 4. Discussion

As an important host of *Ophiocordyceps sinensis*, *Thitarodes xiaojinensis* has not been sufficiently explored in nature. Previous transcriptomic and metabolomic studies have illustrated the morphological characteristics of *O. sinensis* during its development in the hemocoel of *T. xiaojinensis* [30]. However, the physiological and pathological responses of *T. xiaojinensis* to *O. sinensis*, especially during fungal BP and BS in larvae, remain unclear. Here, we investigated the molecular changes of the host infected with *O. sinensis* by using a combined transcriptomic and metabolomic approach.

After *Beauveria bassiana* or *Metarhizium* spp. invasion, PRRs like β-1,3-glucan recognition proteins (βGPR1) and immulectins, signal modulators like serine proteases, signal transduction molecules like Toll ligands, and immune effectors like AMPs and prophenoloxidase could be activated to help insects defend against the infection [44,45,46,47,48]. This study indicated that the upregulated genes in Tx-BP were mainly confined to PGRPs and AMPs. Our previous study showed that a protective layer existed on the cell wall of *Ophiocordyceps sinensis,* avoiding βGPR1 induction [9]. Consistent with this finding, changes in βGPR1 expression were not detected in the fat body of Tx-BP. Upregulation of PGRPs generally leads to nuclear translocation of the transcription factors in the Toll and IMD pathways, which in turn induces the expression of various immune effectors, including AMPs [49]. However, signal transduction molecules in the immune pathways showed no significant change in Tx-BP. This may result from feedback regulation of negative regulators to prevent excessive immune responses and energy consumption, and to avoid collateral damage to the host. For example, serpins can interact with serine proteases, preventing signal amplification, and then inhibit immune responses [50]. TRINITY_DN3553_c0_g2, encoding a serpin, increased by 15-fold in Tx-BP and might play a role in the feedback regulation. Indeed, immune response is a complex process. The precise mechanisms can vary among different insect species. We cannot rule out that *Thitarodes xiaojinenesis* may exploit an uncommon pathway to control the expression of immune-related molecules.

Transcriptome analyses of Tx-BP showed an upregulation of transcripts involved in immunity in response to *Ophiocordyceps sinensis* infection. In Tx-BS, the upregulated genes were mainly enriched in metabolic processes and the oxidative phosphorylation pathway. It is suggested that there is an activation of protein synthesis, metabolism and energy production during this period. At the early phase of infection the concentration of BP is low. Furthermore, the growth of *O. sinensis* blastospores was too slow to compete with other quickly growing infectious microbes. For example, *Isaria farinosa* (Hypocreales: Cordycipitaceae), a broad-spectrum entomopathogenic fungus, could infect and cause the death of the ghost moth larva in only two weeks at the same temperature (our observation), preventing *O. sinensis* from completing its development. Upregulated PGRPs and AMPs in the host may function in recognizing and resisting coinfection with other microbes and ensure relatively safe conditions for the growth of *O. sinensis*. Upon entering the BS stage, the blastospores accumulated to ~6 × 10^8^ cells/mL [8], which is an absolute advantage in number. It would not be necessary to constantly activate the host immune system at this stage, and is consistent with the normalized immune activity of Tx-BS. Additionally, *O. sinensis* upregulated its own antibiotic biosynthesis at the prehyphae and hyphae stages, which was presumed to compensate for the loss of host immunity due to host death [30]. Overall, in this *Thitarodes xiaojinensis*-*O. sinensis* parasitic system, it is more likely that *O. sinensis* actively modulates the immune system of *T. xiaojinensis* to best facilitate its survival in host hemocoel. One option is to avoid activating the host immunity against fungus itself; the other option is to utilize the host immunity to resist other microbes’ co-infection. It seems contradictory, but *O. sinensis* makes it work in some sense. 

Changes in hemolymph composition reflect morphogenetic and biochemical transformations occurring in insect tissues [51]. Our metabolomic data showed a decrease in the level of amino acids and lipids in the hemolymph of Tx-BP and Tx-BS. Another metabolic analysis demonstrated that *B. bassiana* infection increased the levels of amino acids and lipids in silkworm larvae hemolymph. The fungal degradation of host proteins resulted in the accumulation of amino acids. The increased levels of lipids in infected larvae could be due to the breakdown of hemocyte cells upon fungal infection [17]. This inconsistent result may be arising from the different pathogenic strategy. If we simply consider insect hemolymph as a nutrient medium, *B. bassiana*, a toxic strategist, releases toxic substances to destroy the normal physiological activity and behavior of the host and kill the host quickly, host nutrient degradation would be the primary event mainly utilized for fungus development, leading to the higher level of amino acids and lipids in host hemolymph. While for *Ophiocordyceps sinensis*, a growth strategist, the need to keep the host maintaining normal physiological activity and behavior for quite a long time, the level of amino acids and lipids in host hemolymph depends on the sum of biosynthesis and consumption. Higher consumption is reflected in increased utilization not only by fungus development, but also by host development and its extra input in immune responding and metabolism. Overall, the reduction in amino acids and lipids in *Thitarodes xiaojinensis* hemolymph may result from relatively higher consumption than from biosynthesize. 

Our analyses revealed that the majority of lipid components experienced an increase in their concentrations in the fat body of Tx-BS, especially for numerous phospholipids and TAGs. It may explain the increased body weight in the infected larvae. The primary sources of energy in insects are glycogen and TAGs [29]. TAG synthesis and mobilization are important in maintaining energy homeostasis. Fat cells store TAG as energy and release them when needed. Our results showed that the levels of TAGs in the host’s fat body of both Tx-BP and Tx-BS were increased in comparison with those in mock samples. We speculated that there was a competition for TAGs between the host itself and the fungus. The infected *Thitarodes xiaojinensis* boost the conversion of diacylglycerols into TAGs by upregulating diacylglycerol o-acyltransferase expression, so that the host’s lost TAGs consumed by the fungus are compensated and energetic homeostasis in the host is maintained. Major metabolic pathways associated with sugar and lipid metabolism are typically highly conserved across taxa [52]. Our data revealed that the expression of genes involved in the glycolysis pathway increased. Meanwhile, many key enzymes in the related pathways of glycerolipid metabolism and fatty acid synthesis were increased synchronously. These results suggested that the induced glycolysis pathway might be necessary for the synthesis of TAGs and glycerolipids. The increased level of lipids in the host’s fat body was proposed to be highly beneficial for the late development of fungus. The information we described above will give a clue in studies about the regulation of host lipid metabolism after fungal infection.

## 5. Conclusions

In this study, the DEGs and differentially expressed metabolites between *Ophiocordyceps sinensis*-infected and uninfected *Thitarodes xiaojinensis* were identified via transcriptomic and metabolomic analyses. The greatest change for Tx-BP is to selectively upregulate immune recognition and effectors. It would be beneficial for the early settlement of *O. sinensis* to avoid or decrease the competition of other microbe coinfections. For Tx-BS, the immune response reverts to normal levels, coincident with the successful settlement of *O. sinensis*. It is suggested that *O. sinensis* could modulate and utilize host immunity to resist other microbe coinfections. The remarkable impact on Tx-BS occurred in pathways associated with energy production and lipid metabolism, including upregulation of oxidative phosphorylation, glycolysis pathway, fatty acid synthesis and glycerolipid metabolism. Remodeling of phospholipids and lysophospholipids were observed in the fat body of Tx-BP, while most lipids were increased in Tx-BS. Overall, *O. sinensis* infection caused an upregulation of TAG and lipids in the fat body of host larvae, and a reduction of amino acids and lipids in hemolymph. This may be due to the imbalance between biosynthesize and consumption through disorders in the expression of genes associated with the corresponding metabolic pathways. The present study provides a foundation for further investigation of the mechanisms of fungus–insect interactions.

## Figures and Tables

**Table 1:** Expression profile of genes encoding the immune effectors of *Thitarodes xiaojinensis.*

Gene Name	TPM				Log2 (Fold Change)	
	MOCK1	Tx-BP	MOCK2	Tx-BS	Tx-BP	Tx-BS
DEF	729.75	18,663.43	1807.60	1097.30	**4.68**	−0.72
Att3	86.30	92.32	577.28	157.19	0.09	−1.88
Att4	521.38	4824.39	894.29	332.45	**3.21**	−1.43
Att5	10.40	27.39	10.82	9.40	**1.40**	−0.20
Cec1	2.63	4.30	8.37	0.81	0.71	−3.37
Cec2	4.22	69.44	23.61	7.38	**4.04**	−1.68
Cec3	0.95	8.30	1.07	0.33	**3.13**	−1.70
Cec5	5.36	65.61	19.32	8.73	**3.61**	−1.15
Gloverin	174.37	5204.20	475.21	609.07	**4.90**	0.36
Lys1	11,787.02	22,424.07	11,140.33	20,480.92	0.93	0.88
Lys2	79.76	418.33	365.93	139.23	**2.39**	−1.39
Lys3	5.13	294.97	11.15	5.06	**5.85**	−1.14
Lys5	57.02	119.74	35.47	140.93	**1.07**	1.99
Lys6	24.71	7.46	14.08	55.34	−1.73	1.98
LLP1	378.07	550.47	878.01	392.43	0.54	−1.16

The fold change was calculated by log2 (TPMtreated/TPMcontrol). Bold font indicates significantly upregulated genes. DEF, defensin; ATT, attacin; Cec, cecropin; Lys, lysozyme; LLP, lysozyme-like protein. TPM, transcripts per million reads; Tx-BP, *T. xiaojinensis* samples collected when *O. sinensis* entered BP stage; MOCK1, the control for Tx-BP; Tx-BS, *T. xiaojinensis* samples collected when *O. sinensis* entered BS stage; MOCK2, the control for Tx-BS.

## Data Availability

The data presented in this study are openly available in https://www.scidb.cn/s/ja63m2, accessed on 20 July 2023.

## Supplementary Materials

The following are available online at https://www.mdpi.com/article/10.3390/microorganisms11092361/s1, Table S1: Summary of sequencing quality; Table S2: The percentage of the sequences annotated in six databases; Table S3: All differentially expressed gene TPM value and gene annotation details; Table S4: Expression profile of genes in Toll, IMD, JNK and JAK/STAT pathway of *Thitarodes xiaojinensis*; Table S5: Oligonucleotide primer sequences used in this study. Figure S1: Principal component analysis of the RNA sequencing data; Figure S2: DEGs distribution between two groups analyzed. The number of DEGs is indicated on the right of the histograms; Figure S3: GO functional classification of differentially expressed genes. The blue bars represent molecular function; green bars represent cellular component and red bars represent biological process functions; Figure S4: PCA plot of the different sample groups (MOCK1, MOCK2, Tx-BP and Tx-BS) and quality control. Each point represents a biological replicate; Figure S5: PLS-DA score plot and validation plots for the metabolic profiling results. The criteria for stability and credibility are as follows: R2Y greater than Q2Y values in score plots; Figure S6: qRT-PCR validation of the RNA-Seq data. The ribosomal protein S3 (rpS3) was used as a reference gene. The bars represent means ± SEM from three independent measurements, while letters on the bars indicate significance levels based on *p* < 0.05; Figure S7: qRT-PCR validation of the gene associated with degradation of glycerolipids and fatty acids. The ribosomal protein S3 (rpS3) used as a reference gene. The bars represent means ± SEM from three independent measurements, while letters on the bars indicate significance levels based on *p* < 0.001.

## Abbreviations

Tx-BP: *Thitarodes xiaojinensis* samples collected when *Ophiocordyceps sinensis* was in BP stage; Tx-BS: *T. xiaojinensis* samples collected when *O. sinensis* was in BS stage; AMP: antimicrobial peptide; BP: blastospores in proliferative stage; BS: blastospores in the stationary stage; PreHy: prehyphae; Hy: Hyphae; cDNA: complementary DNA; bp: base pair; TPM: transcripts per million reads; DEGs: differentially expressed genes; FDR: false discovery rate; TAGs: Triglycerides; GO: Gene Ontology; KEGG: Kyoto Encyclopedia of Genes and Genomes; LC-MS/MS: Liquid chromatography-tandem mass spectrometry; COG: Clusters of Orthologous Groups of proteins; ESI: electrospray ionization; HMDB: human metabolome database; PCA: principlal component analysis; PLS-DA: partial least squares discriminate analysis; VIP: variable importance in the projection; FC: fold change; QC: quality control; qRT-PCR: Quantitative real-time polymerase chain reaction; rpS3: ribosomal protein S3; ANOVA: one-way analysis of variance; COG: Clusters of Orthologous Groups of proteins; NR: NCBI non-redundant protein sequences; SwissProt: a manually annotated and reviewed protein sequence database; Pfam: Protein family; PCA: Principal component analysis; IMD: immune deficiency; AGE-RAGE: advanced glycation end products (AGEs) interact with the receptor for AGEs; ECM: extracellular matrix; DEGs: differentially expressed genes; DEMs: differentially expressed metabolites; LPC: lysophosphatidylcholines; LPA: lysophosphatidic acids; LPE: lysophosphatidyl ethanolamines; PC: phosphatidylcholine; PA: phosphatidylethanolamine; PS: phosphatidylserine; gapdh: glyceraldehyde-3-phosphate dehydrogenase; pgm: phosphoglucose mutase; eno: enolase; pk: pyruvate kinase; pc: pyruvate carboxylase; cs: citrate synthase; atpcl: ATP citrate lyase citrate; gpdh: glycerol-3-phosphate dehydrogenase; gk: glycerol kinase; gpat: glycerol-3-phosphate acyltransferase; agpat: 1-acyl-sn-glycerol-3-phosphate acyltransferase; dgat: diacylglycerol o-acyltransferase; GK: glycerol kinase; aCs: acyl-CoA synthetase; cOa: carnitine O-acetyltransferase; aCd: acyl-CoA dehydrogenase; eCh: enoyl CoA hydratase; 3-hCd: 3-hydroxyacyl CoA dehydrogenase; 3-kCtB: 3-ketoacyl-CoA thiolase B; JNK: c-Jun N-terminal protein kinases; JAK/STAT: janus kinase/signal transducer and activator of transcription; PAMPs: pathogen-associated molecular patterns; PPRs:pattern recognition receptors; PGRP: peptidoglycan recognition proteins.
