# Supplementary material for: Transcriptome and Metabolome Analyses of Thitarodes xiaojinensis in Response to Ophiocordyceps sinensis Infection
Source: Microorganisms. 2023 Sep 21;11(9):2361. doi: 10.3390/microorganisms11092361 (PMC10537090; doi:10.3390/microorganisms11092361)
Supplement: Supplementary file 1 [file microorganisms-11-02361-s001.zip › Supplementary file S2.pdf]

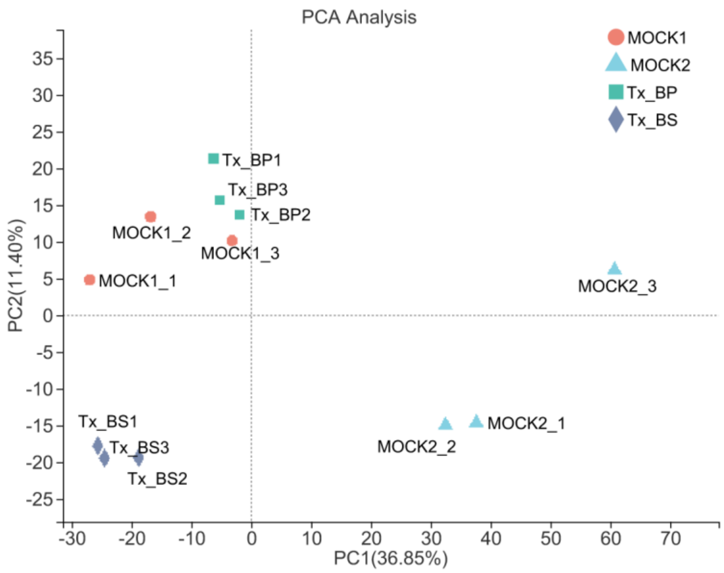

Figure S1: Principal component analysis of the RNA-Sequencing data.

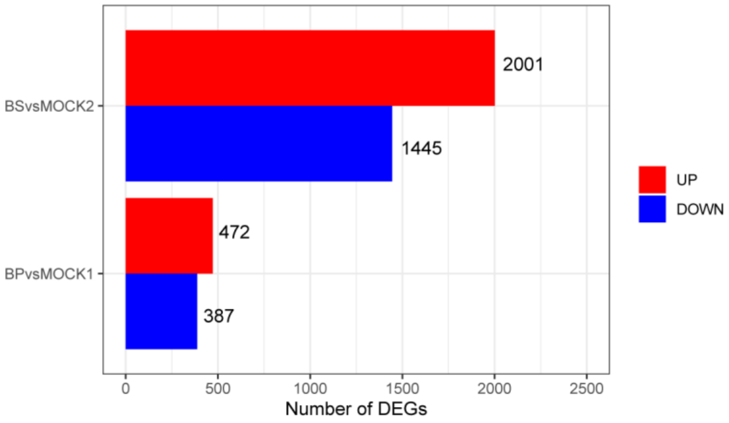

Figure S2: DEGs distribution between two groups analyzed. The number of DEGs is indicated on the right of the histograms.

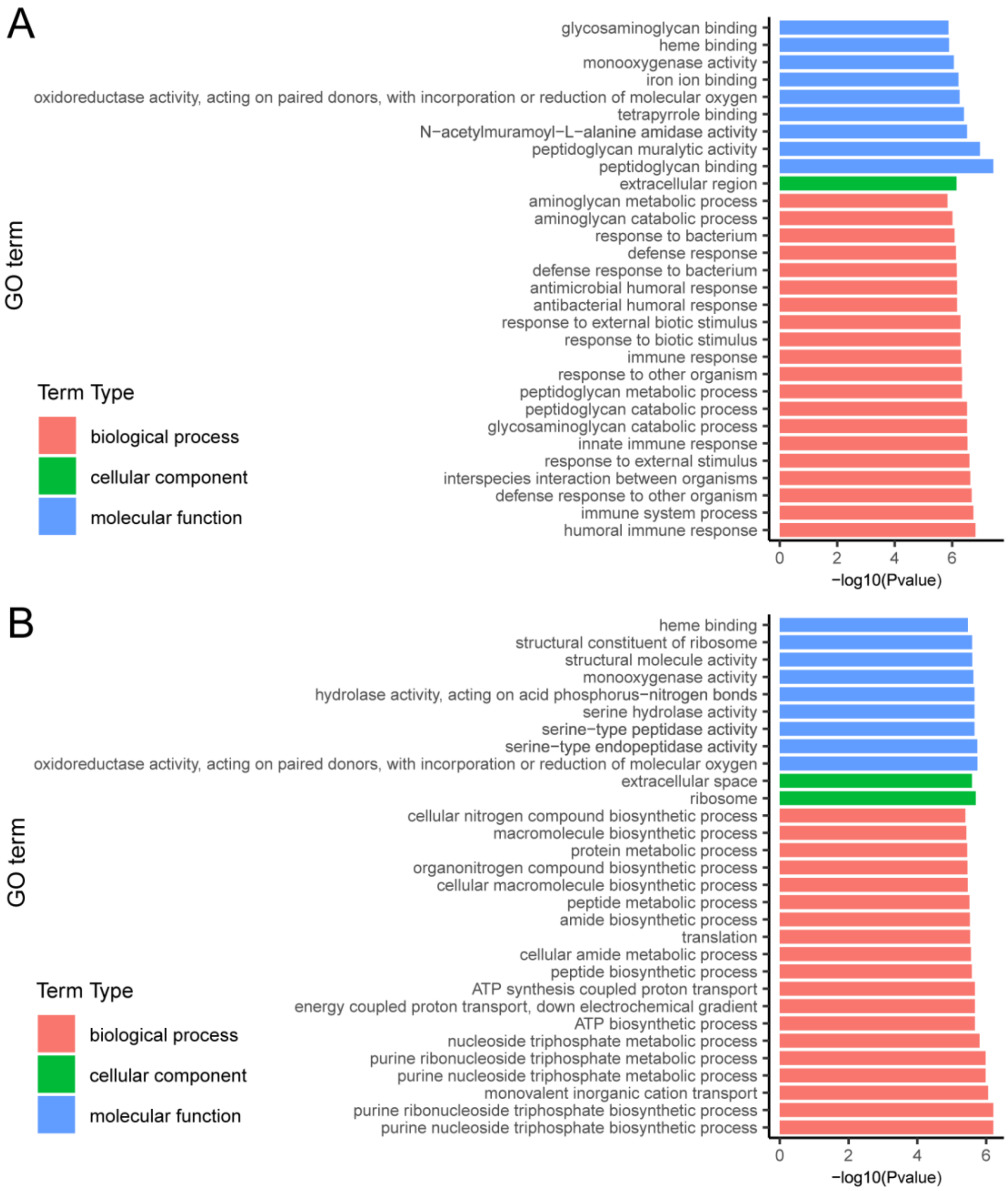

Figure S3: GO functional classification of differentially expressed genes. The blue bars represent molecular function; green bars represent cellular component; red bars represent biological process functions.

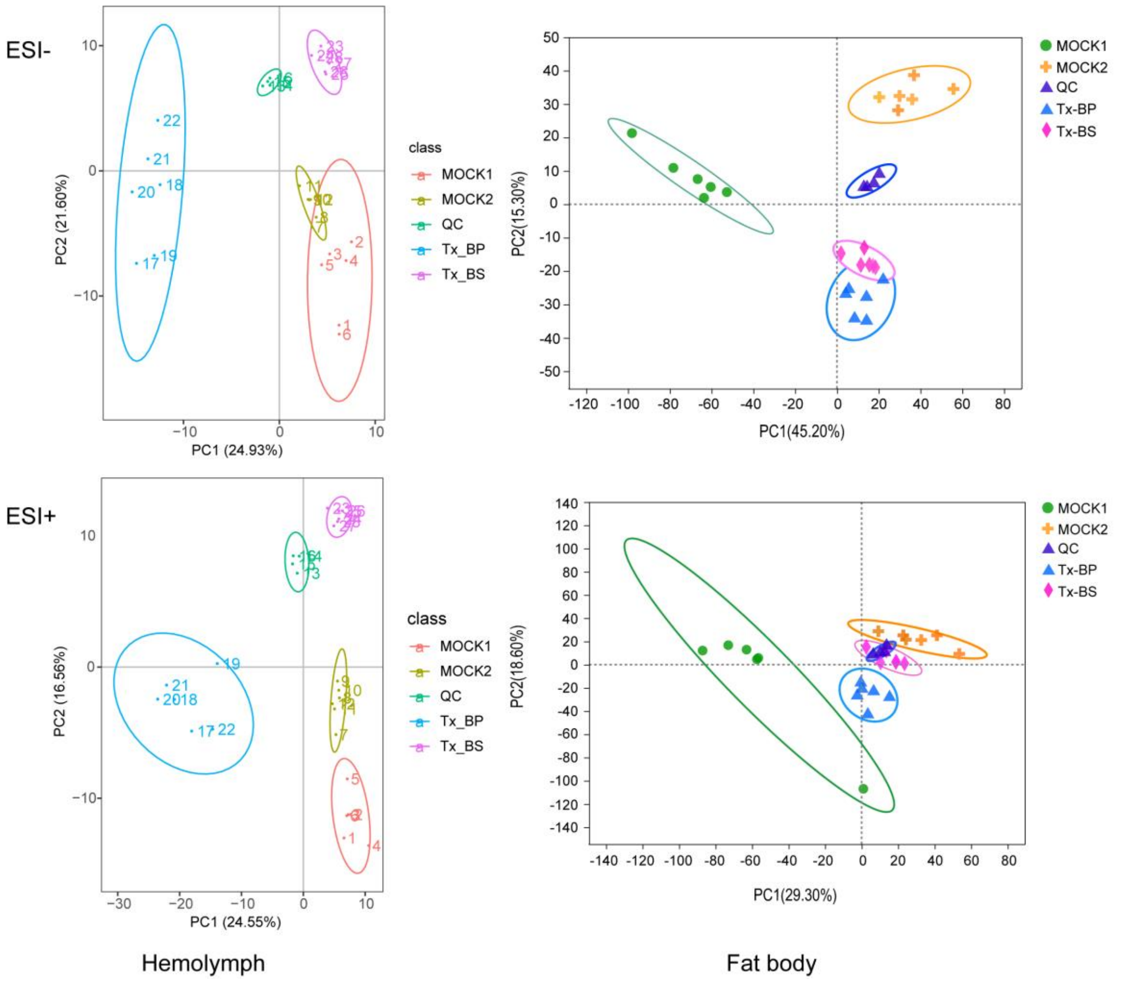

Figure S4: PCA plot of the different sample groups (MOCK1, MOCK2, Tx-BP, Tx-BS) and quality control. Each point represents a biological replicate.

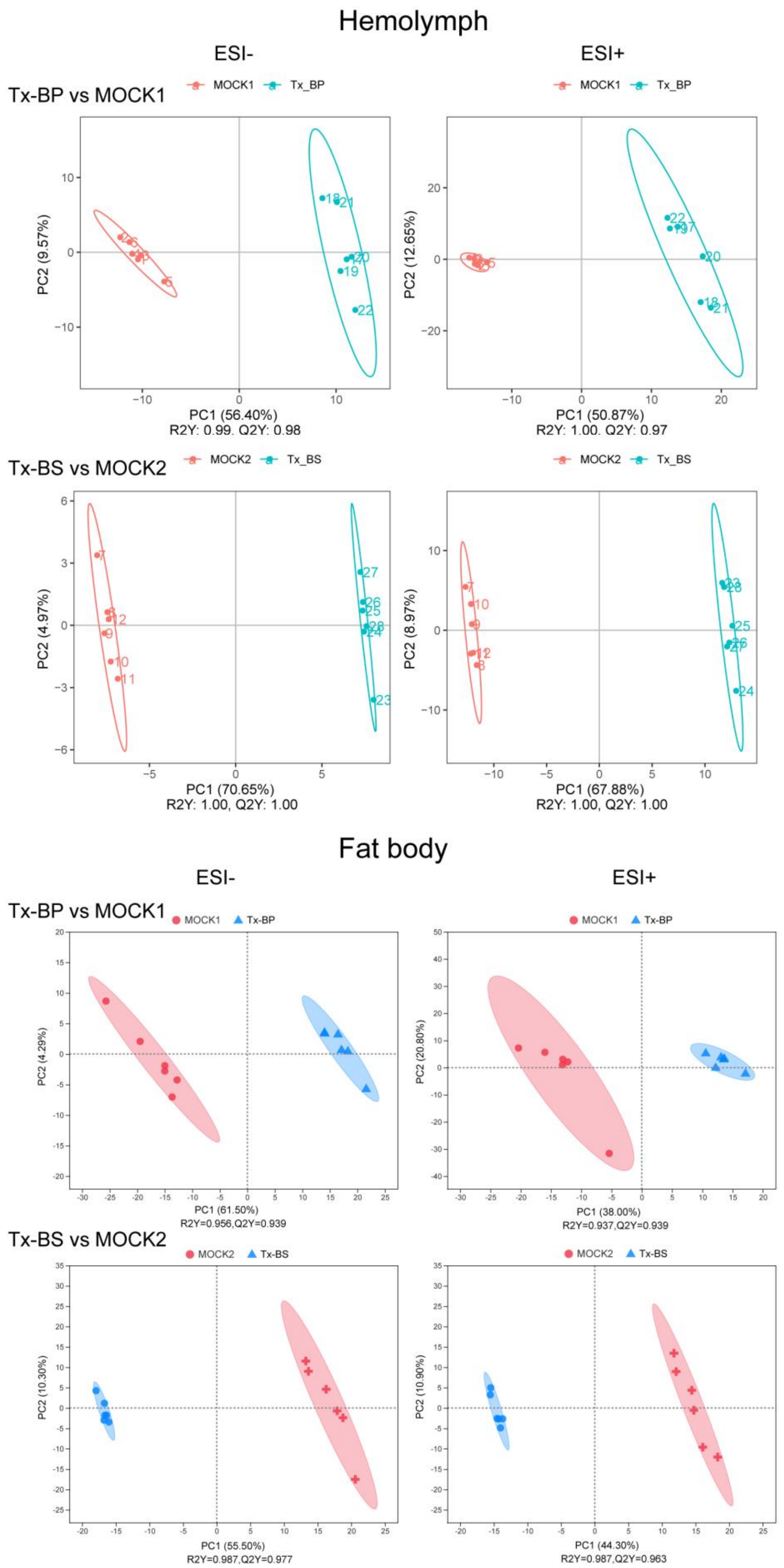

Hemolymph
ESI-
ESI+
Tx-BP vs MOCK1
MOCK1
Tx_BP
PC1 (56.40%)
PC2 (9.57%)
R2Y: 0.99, Q2Y: 0.98
PC1 (50.87%)
PC2 (12.65%)
R2Y: 1.00, Q2Y: 0.97
Tx-BS vs MOCK2
MOCK2
Tx_BS
PC1 (70.65%)
PC2 (4.97%)
R2Y: 1.00, Q2Y: 1.00
PC1 (67.88%)
PC2 (8.97%)
R2Y: 1.00, Q2Y: 1.00
Fat body
ESI-
ESI+
Tx-BP vs MOCK1
MOCK1
Tx-BP
PC1 (61.50%)
PC2 (4.29%)
R2Y=0.956,Q2Y=0.939
PC1 (38.00%)
PC2 (20.80%)
R2Y=0.937,Q2Y=0.939
Tx-BS vs MOCK2
MOCK2
Tx-BS
PC1 (55.50%)
PC2 (10.30%)
R2Y=0.987,Q2Y=0.977
PC1 (44.30%)
PC2 (10.90%)
R2Y=0.987,Q2Y=0.963

Figure S5: PLS-DA score plot and validation plots for the metabolic profiling results. The criteria for stability and credibility are as follows: R2Y greater than Q2Y values in score plots.

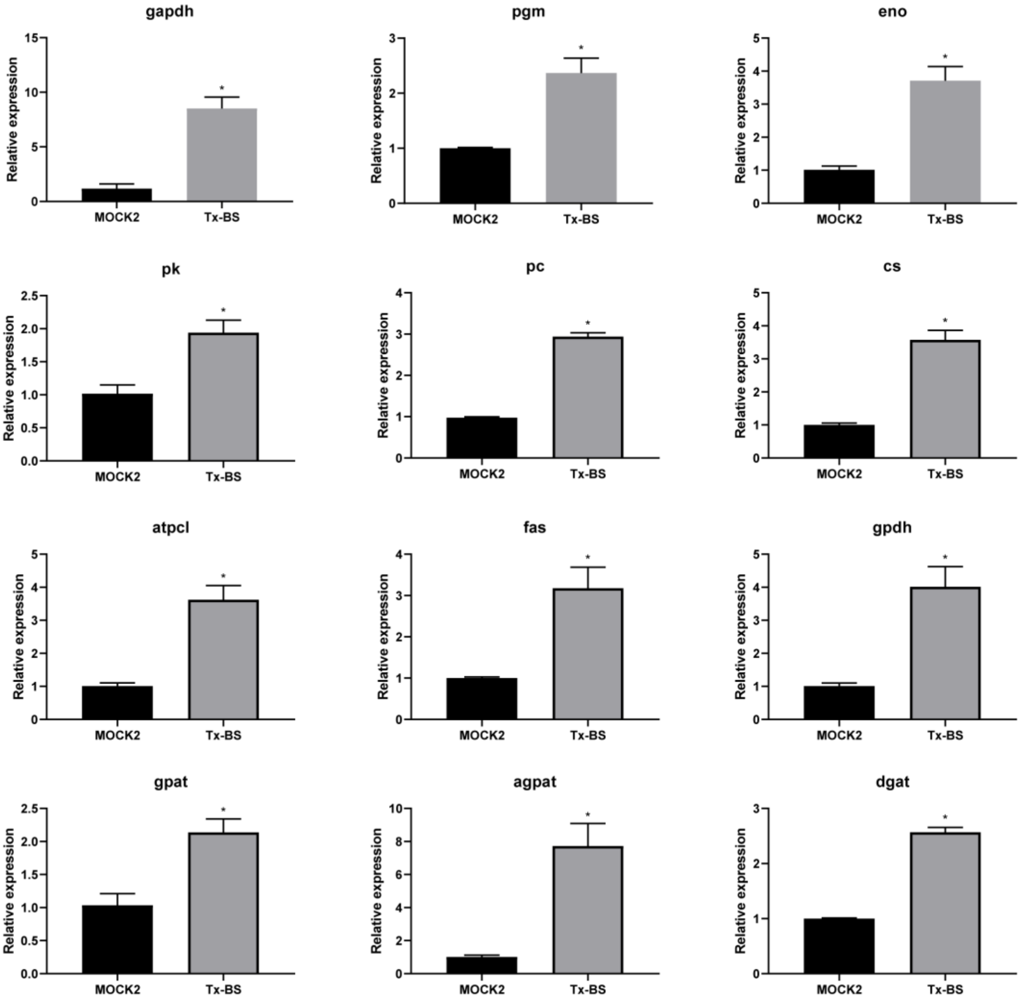

Figure S6: qRT-PCR validation of the RNA-Seq data. The ribosomal protein S3 (rpS3) used as a reference gene. The bars represent means ± SEM from three independent measurements, while letters on the bars indicate significance levels based on $p < 0.05$.

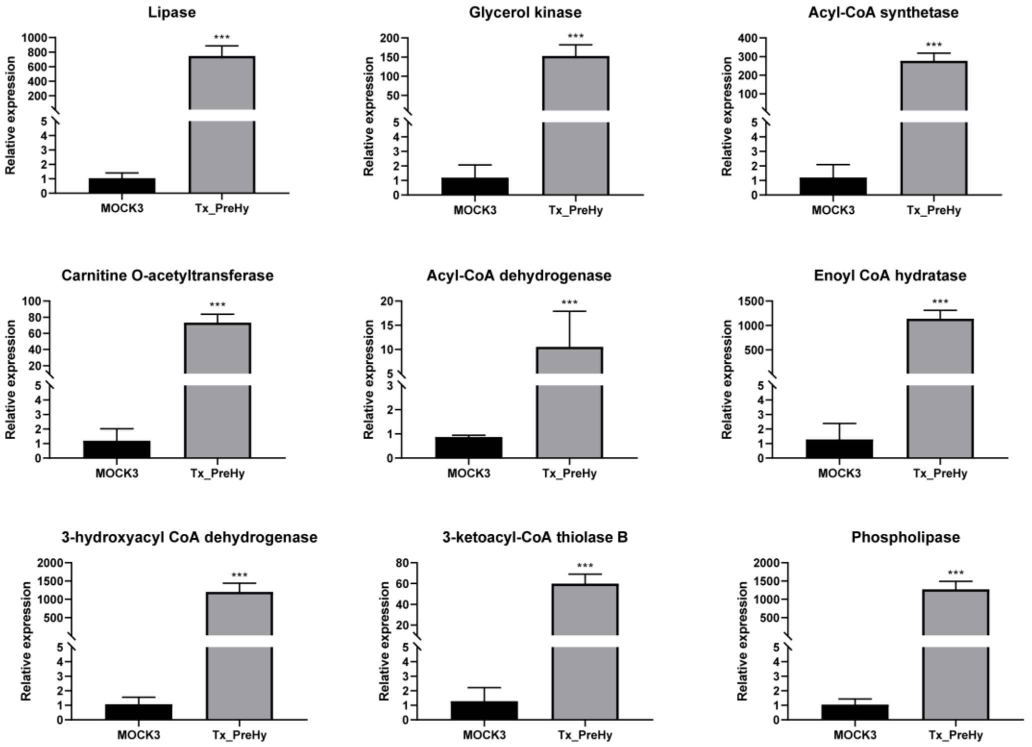

Figure S7: qRT-PCR validation of the gene associated with degradation of glycerolipids and fatty acids. The ribosomal protein S3 (rpS3) used as a reference gene. The bars represent means ± SEM from three independent measurements, while letters on the bars indicate significance levels based on $p < 0.001$. Tx-PreHy, *T. xiaojinensis* samples collected when *O. sinensis* entered PreHy stage; MOCK3, the control for Tx- PreHy.
